# Relationship of Allergy with Asthma: There Are More Than the Allergy “Eggs” in the Asthma “Basket”

**DOI:** 10.3389/fped.2017.00092

**Published:** 2017-04-28

**Authors:** George V. Guibas, Alexander G. Mathioudakis, Marina Tsoumani, Sophia Tsabouri

**Affiliations:** ^1^Division of Infection, Immunity and Respiratory Medicine, University Hospital of South Manchester, University of Manchester, Manchester, UK; ^2^Child Health Department, School of Medicine, University of Ioannina, Ioannina, Greece

**Keywords:** asthma, endotype, phenotype, wheeze, pediatrics, classification, allergy

## Abstract

Asthma and allergy share a similar and very close course, especially through childhood. Considerable research effort has been put in untangling these associations; however, it is now becoming obvious that this is an exceedingly difficult task. In fact, each research breakthrough further perplexes this picture, as we are steadily moving toward the era of personalized medicine and we begin to appreciate that what we thought to be a single disease, asthma, is in fact an accumulation of distinct entities. In the context of this “syndrome,” which is characterized by several, as of yet poorly defined endotypes and phenotypes, the question of the link of “asthma” with allergy probably becomes non-relevant. In this review, we will revisit this question while putting the emphasis on the multifaceted nature of asthma.

## Introduction

Asthma is a chronic pulmonary inflammatory disease wherein the innate and adaptive immune systems cooperate with epithelial cells to cause airway hyperresponsiveness (AHR), mucus overproduction, airway wall remodeling, and bronchoconstriction. Clinically, it is characterized by recurrent episodes of wheezing, breathlessness, and chest tightness. It affects more than 10% of the population in many westernized countries and more than 300 million people worldwide (1). It has a great impact in childhood and is the leading cause of school absenteeism in the United States, causing approximately 50% of children to miss at least one school day yearly (1).

Asthma is seen as an allergic disease; this assertion, although well documented, is probably an oversimplification. In fact, up to the last decade, our view of asthma as a single disease was likely oversimplified. Although main characteristics of asthma are airflow obstruction, bronchial hyperresponsiveness, and underlying inflammation, it is rare that all these characteristics can be found in all patients of large cohorts. Asthma recently started to be recognized as a “syndrome,” a complex condition with variability in its pathophysiology, severity, natural history, comorbidities, and treatment response (2). Therefore, an important question in the last decade is whether asthma is a single disease with a variable presentation, or several “linked” diseases that share salient clinical features (3, 4). It is now becoming clear that the latter is probably true and that the diagnostic label “asthma” likely encompasses many different disease variants (phenotypes) with different etiologies and underlying pathophysiological mechanisms.

It is this complexity of asthma that makes it difficult to discern its link with allergy (atopy). These are indeed two intertwined conditions and are closely associated in the minds of most clinicians. However, several asthma subtypes may in fact have little to do with atopy. This is where the narrative about the asthma endotypes and phenotypes, and our need to move into a new era of personalized medicine, comes into place. About a decade ago, the word “phenotype” entered the field of asthma research and management (2). In scientific language, the word “phenotype” refers to “the observable properties that an organism displays in the context of a certain disease, which are caused by the interactions of the organisms genotype with the environment.” In simpler terms, a particular phenotype (and in this case an asthma phenotype) is defined and told apart from the other asthma phenotypes by its prominent/unique clinical characteristics (5). Once the discussion about the phenotypes was afoot, it became glaringly obvious that these subtypes of asthma were often underpinned by discreet pathophysiological processes, giving rise to the concept of “endotypes” and increasing asthma complexity. Hence, the term “endotype” defines a specific biological pathway that underpins the clinical observations, which constitute a phenotype (2, 6).

**Figure 1:**
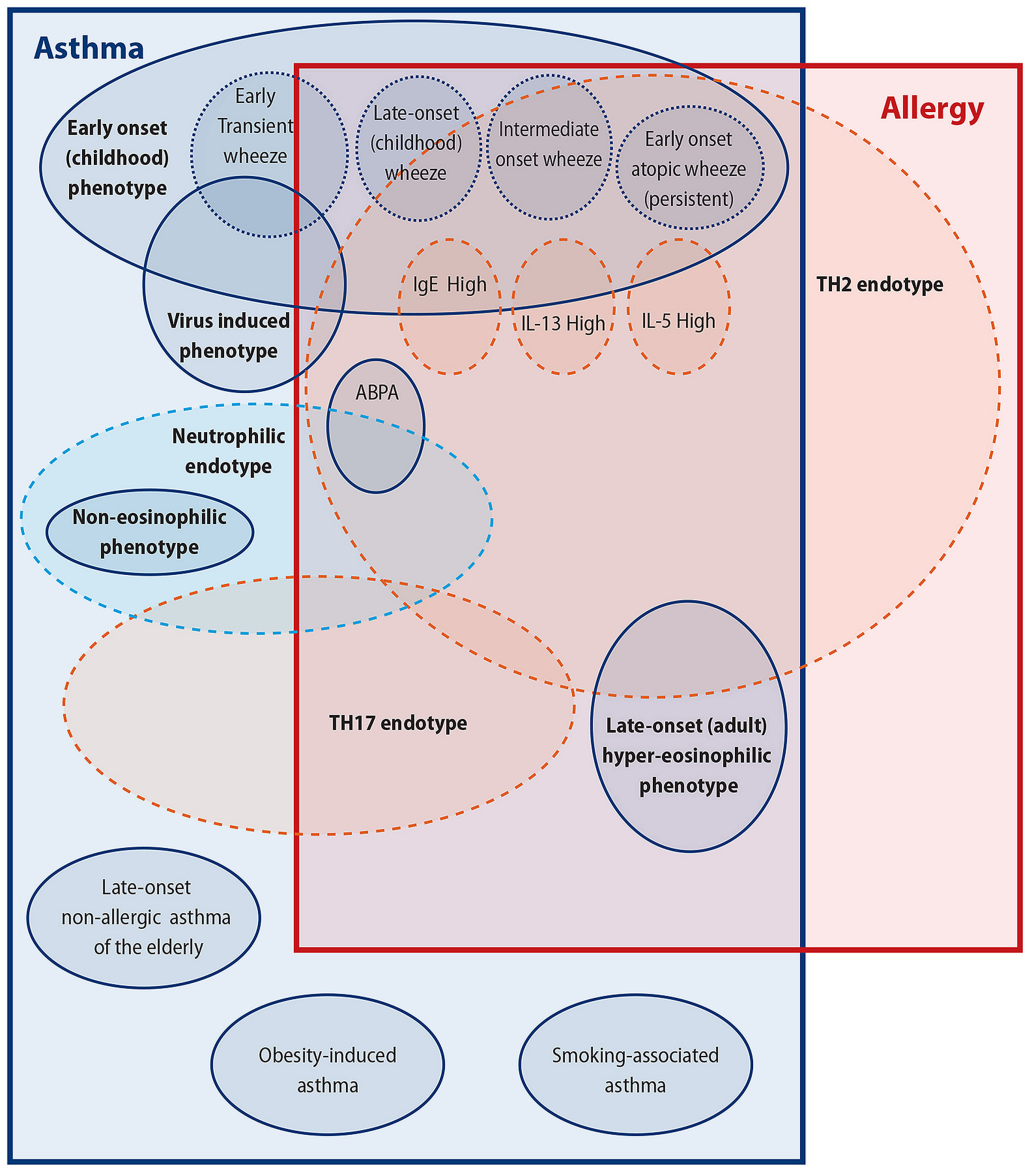
**Attempting to untangle the asthma–allergy associations**. There are more than the allergy “eggs” in the asthma “basket.”

When one looks from this perspective, it is easy to see the reasons for the difficulty to clarify the link of allergy/atopy to asthma; not least important of these reasons being that asthma may in fact include several different clinical conditions underpinned by several different pathophysiological processes. In this review, we will try to look into this asthma/atopy relationship from the perspective of the complex multifaceted disorder, that is asthma, and will attempt to individually link some of its subparts with atopy (Figure 1). This is, however, unlikely to give us a clear picture of the relationship of asthma with allergy; in our opinion, this relationship is oversimplified and is therefore likely to be wrong as was our view of an oversimplified asthma disease for many decades now.

## Asthma Endotypes and Allergy

The expression “endotype” refers to “an asthma subtype defined by a distinct functional or pathophysiological mechanism” (2), it is a widely used and discussed term, and little consensus exists about endotype numbers and characteristics. Currently, standardized diagnostic/management approaches based on endotypes are being sought (2), and some are being used in the clinical setting (e.g., omalizumab for IgE-high allergic asthma), but the majority of asthma cases are still being treated with the one-size-fits-all management, which was established decades ago.

### Th2 Endotype

This is one of the widest and probably the best-defined asthma endotypes thus far. It is characterized by a type 2 immune response that involves Th2 cells, B cells, basophils, eosinophils, mast cells, major cytokines secreted from immune cells [interleukin (IL)-4, IL-5, IL-9, and IL-13], and others secreted from epithelial cells (IL-25, IL-31, IL-33, and TSLP) (7). This is the endotype underpinning allergic asthma and is strongly linked to atopy, IgE production, and eosinophilic inflammation (8). This is arguably one of the most important endotypes in childhood, as it is closely associated with the early-onset asthma (EoA) phenotype that usually starts during childhood and early adolescence. This phenotype, described in detail in the next paragraphs, consists of several other subphenotypes, has a high prevalence, and often persists into adulthood. This endotype is indeed prevalent in adulthood, seeing that about 50% of *mild* asthmatics have an endotype that is associated with eosinophilia, mast cell activation, development of allergen-specific IgE, and Th2 cytokine production (9). Several subendotypes might exist within this endotype, such as the IL-5-high, IL-13-high, or IgE-high (10).

Given this endotype’s dependence on Th2 cytokines, it is unsurprising that it responds to therapies targeting IL-4 and IL-13, particularly in allergen-challenge models (8, 11, 12). Indeed, IL-4/13 blockade is most efficacious in patients with Th2-related asthma, especially with peripheral eosinophilia (13–15). Anti-IL-5 treatment in some patients has been associated with lower frequency of acute asthma, a steroid-sparing effect, and improved lung function (16–18). Regulators of Th2-type cytokines (such as IL-25, IL-33, and TSLP) and inhibitors of TSLP are gaining ground (19). However, one must recognize that a major pathogenic pathway such as the Th2 endotype is complex and heterogeneous, with several determinants that have non-linear dynamic interactions (10, 20). Therefore, currently, there is only one routinely used biologic therapy for Th2 asthma, omalizumab, an anti-IgE molecule; In any case, this further documents the close link of this endotype with allergy (19). There is now considerable experience with this drug, which works best in atopic patients with severe, inadequately controlled disease (21).

### Non-Th2 Endotype

Approximately half of asthmatic patients have Th2-no/Th2-low endotype (15), yet much less is known about this heterogeneous group. Some agents of interest are the cytokines IL-17, IL-1b, TNF-a (22), and a chemokine receptor (CXCR2), which are associated with neutrophilic inflammation (10). From the clinical point of view, these patients show less airway obstruction and hyperreactivity than Th2-high asthmatics. Importantly, these are generally non-atopic-patients, and there is little to no evidence of allergy in childhood or beyond (23).

Two major mechanisms leading to non-type 2 asthma have been postulated: (i) activation of the IL-17-dependent pathway and (ii) innate immune response dysregulation bringing about neutrophil inflammation (20).

### Th17 Endotype

Th17 cells are characterized by the production of IL-17A, IL-17F, and IL-22. They develop in response to transforming growth factor-b and IL-6 production and are dependent on the expression of transcription factor RORgt. Accumulating evidence suggests a role for TH17 cells in asthma, especially severe steroid-resistant asthma. Increased IL-17A+ cells can be found in lung biopsies of patients with severe asthma compared to those with mild (24). In both adults and children, serum IL-17A is significantly higher in severe asthmatics compared to mild ones (25), and it has been linked to remodeling and AHR. This asthma endotype has little relation with atopy.

### Neutrophil Endotype

Neutrophil inflammation is well established in mouse models of asthma, where it has been linked to the development of airway hyperresponsiveness (AHR) and remodeling. In an experimental model of Th1/neutrophil-predominant asthma, TNF-a reduced the responsiveness to steroids, whereas its neutralization restored steroid responsiveness (26). In humans, patients with non-allergic asthma demonstrated considerable neutrophil inflammation induced by IL-17-shifted pro-inflammatory immune reactions (7). A role for a dysregulated innate immune response in neutrophilic asthma has been proposed; this is characterized by altered gene expression of toll-like receptors, and increased expression of genes linked to IL-1b and TNF-a/nuclear factor-kB (27). Enhanced neutrophil chemotaxis/survival in the airways and impairment of anti-inflammatory mechanisms could further underlie this endotype. Obviously, there is little room for an important atopy component in this endotype.

### Mixed Th2/Th17 Endotype

There exists a mixed Th17/Th2 endotype in asthma, as Th2 cells can differentiate into dual-positive Th2/Th17 cells (28). These cells were identified in the bronchoalveolar fluid of asthmatic patients (29).

The relationship between the Th17 and Th2 responses is highly complex. IL-17 produced in response to injured epithelium could enhance the production of IL-4 and IL-13 from Th2 cells (7). Conversely, IL-4 and IL-13 may amplify Th17 responses by upregulating CD209a expression on dendritic cells (7). In any event, this mixed endotype has seen little research, and although it is likely associated with allergy, this remains to be elucidated.

## Asthma Phenotypes and Allergy

### Early-Onset Asthma

The early asthma phenotype is prone to eczema development in early childhood (30). Eczema implies that this phenotype is mostly Th2 driven. A Th2 association in EoA is described (31, 32), but cases with low IgE and limited response to inhaled steroids suggest that there are forms of EoA not related to Th2, exemplified by virus-induced wheeze (33).

Early-onset asthma can be classified into several subphenotypes with varying association to atopy. The Tucson population-based birth cohort study retrospectively subclassified preschool wheeze into three groups: “transient wheeze,” “early persistent wheeze,” and “late-onset asthma” (34). Transient wheeze is thought to be caused by viral infections. Viral and allergy mechanisms could also cooperate to orchestrate the development of both transient wheeze and future allergic asthma (35). Early infection with respiratory syncytial virus has been found to increase the susceptibility to allergic asthma *via* the IL-4 receptor pathway (36). Another subphenotype is “early-onset allergic asthma” (represented in the Tucson study by “early persistent wheeze”), the classic form of persistent asthma that has a childhood onset and bares allergic features, including allergen sensitization and allergic rhinitis. Airway eosinophilia is common in early-onset allergic asthma, and a TH2-dominant inflammatory process is believed to underlie it. Allergy involvement is vital as inhalation of a specific allergen triggers bronchoconstriction and inflammatory cell influx. The efficacy of omalizumab, and the studies on IL-4/IL-13 modifiers, implies a central role of IgE and TH2 cells/cytokines in this subphenotype (2).

Other subphenotypes of this variant have also been recognized. Four subtypes have been identified by unsupervised cluster analyses on 161 subjects in the pediatric asthmatic cohort from the Severe Asthma Research Program (SARP) (37). Cluster 1 consists mainly of mild, later onset, and less atopic asthma with normal lung function. The other clusters represent the early-onset, atopic asthma subphenotype, with, however, variable severity and lung function. These clusters were similar to the ones seen in the adult SARP analyses (11, 38), where the subphenotype of “early-onset, atopic asthma” represented the majority of cases reported. Other cases included “obesity-induced asthma” and “late-onset (adult) non-atopic asthma” of varying subphenotypes.

Other well-known phenotyping attempts include The Avon Longitudinal Study of Parents and Children, which collected data on wheeze at multiple time points from birth to age 7 years, for 6,265 UK children (39). A distinct new phenotype was identified, “intermediate onset wheeze” (onset at 4–6 years of age), which showed the strongest associations with atopy. Late-onset wheeze was also strongly associated with cat, house dust mite, and grass pollen sensitization. “Early-onset atopic asthma” (which in this cohort was likely represented by the “persistent wheeze” cases) had an onset of 6–18 months of age and was also strongly associated with atopy (39). These findings regarding the wheeze subphenotypes and their relation with atopy were similar to those from analyses of the Dutch Prevention and Incidence of Asthma and Mite Allergy study, a multicenter birth cohort that enrolled 4,146 pregnant women (40). Other findings from a population-based longitudinal cohort that enrolled 1,650 preschool children in the UK were largely similar although the authors failed to detect an “intermediate onset wheeze” subset and noted a “non-atopic, persistent wheeze” subset (41). This subset was also detected in an independent population-based cohort by the same authors (validation cohort) (42) and could partly represent the “late-onset wheeze” subset reported in the other studies.

Early transient wheeze is largely underpinned by the virus-induced asthma phenotype. Virus-induced asthma is a very common phenotype in children, and it has been very well described as the “September epidemic” (43). Although “virus-induced asthma” is—as the name of the sub-phenotype indicates—“transient,” virus infections can also alter the course of preexisting asthma or can modify the immune system, hence increasing susceptibility to allergen sensitization and/or asthma in childhood (44). Indeed, wheezy episodes associated with rhinovirus are a strong predictor of asthma (45). This is another testament to the close association of the pediatric asthma subphenotypes with allergy, as even this variant, which is mainly induced by viruses, can be affected by individual atopic status.

### Adult-Onset (Severe) Hypereosinophilic Asthma

Adult-onset asthma with highly elevated numbers of eosinophils is associated with Th2 cytokines and Th2 inflammatory cells like eosinophils, mast cells, and basophils. Eosinophil numbers between 150 and 300 eosinophils per milliliter have been used in asthma trials to define hypereosinophilic asthma, but full consensus is still lacking. The patients often show severe reactions after cyclooxygenase inhibitor intake. However, regardless of the eosinophil basis, the link of this phenotype with the Th2 endotype is not clear (46). This is another poorly defined phenotype as there is a group of individuals who demonstrate strong eosinophilic inflammation but a paucity of symptoms (47). Compared to early-onset Th2 asthma, adult-onset asthma is characterized by the presence of raised eotaxine-2/CCL24 levels; eotaxine-2/CCL24 is a potent proeosinophilic chemokine, which might be the cause of the raised eosinophil count (48). The link of this phenotype with atopy is not clear, but given the eosinophil component, one can assume that some association may exist. Such a link is also supported by the responsiveness of patients with hypereosinophilic, adult-onset asthma to IL-5-targeted therapies (16).

### Late-Onset Non-allergic Asthma of the Elderly

Asthma in the elderly, above the age of 65, is another phenotype (49). Elderly patients with asthma tend to be more symptomatic, with more pronounced airway obstruction, frequent hospital admissions, and a significantly higher mortality (50). They also have frequent comorbidities, which contribute to the severity of the disease. While asthma is prevalent in these ages [6.9% of people above the age of 65 in USA (51)], it is significantly underdiagnosed. This phenotype can be divided into persistent asthma that started early in life and newly diagnosed asthma. Neutrophilic airway inflammation is more prevalent among elderly patients, while atopy and elevated IgE levels are less frequent, but this largely depends on the “new-onset *vs* persistent asthma from a younger age” distinction.

### Obesity-Related Asthma

Another no-/low-Th2 endotype has been identified in obese asthmatics (52). Obesity-induced asthma is characterized by lack of atopy, female predominance, and late onset (53). It is generally more severe and harder to control as it is less responsive to standard controller therapies. Also, large observational studies have demonstrated that obesity is associated with wheezing even in non-asthmatic individuals (54). Asthma is significantly overdiagnosed in these patients, who do not gain benefit from anti-inflammatory asthma treatment. Non-asthma wheezing has complicated research into the obesity-related phenotype, and it still remains to be elucidated whether this phenotype is underpinned by several endotypes or a single endotype with defined inflammatory pathways. In any case, atopy probably has little involvement here.

### Non-Eosinophilic Asthma

Non-eosinophilic asthma is a well-documented and prevalent asthma phenotype, as approximately half of asthma patients have no evidence of eosinophilic inflammation (55). Neutrophilic inflammation has been identified in several studies as a hallmark of this variant (e.g., the SARP cohort). Furthermore, non-eosinophilic asthma is associated with innate immune dysfunction and increased expression of several biomarkers, including neutrophil elastase, TLR2 and 4, IL-1β, IL-8, MMP-9, and TNF-a (56). As expected, the response of non-eosinophilic asthma to inhaled corticosteroids is weak, and its management can be challenging. No consensus exists about the neutrophil cell count threshold that could define neutrophilic asthma. A relationship of this phenotype with smoking is discussed. It is unlikely that this phenotype is strongly connected to atopy (57).

### Smoking-Associated Asthma

In a recent cluster analysis, the phenotype of smoking-related asthma was described (51). This group consisted of mainly male adults (66%) who are non-atopic. They had preserved postbronchodilation spirometry but more pronounced respiratory symptoms. Cigarette smoking is a well-known aggravating factor of asthma symptoms and associated burden. Most likely, coexistence of early changes of chronic obstructive pulmonary disease (COPD) also contributes to the clinical presentation of this phenotype, as it has been demonstrated that clinical symptomatology of COPD could precede significant pulmonary function decline. Therefore, this phenotype probably corresponds to asthma-COPD overlap syndrome.

### Allergic Bronchopulmonary Aspergillosis (ABPA)

Allergic bronchopulmonary aspergillosis is a well-described hypersensitivity condition following colonization of the airways by *Aspergillus fumigatus*. This asthma endotype is characterized by a mixed pattern of neutrophilic and eosinophilic airway inflammation, elevated *Aspergillus*-specific IgE and IgG (58). Given this pathophysiological mechanism, ABPA could be considered an allergic endotype.

## Conclusion

We are entering an era where precision medicine is gaining considerable ground, especially where complex diseases come into play. These diseases ideally exemplified by asthma had been tackled for decades with a one-suits-all management, only to be noted now that this approach leaves much to be desired in terms of efficacy, in a large portion of patients. This group that fails to respond to the conventional standardized approach accounts for more than 50% of asthma-related health-care utilization and is at increased risk of death due to asthma (7). Therefore, it is imperative that such patients who may benefit from a more personalized therapeutic approach are recognized (22).

In this context, categorization of patients *via* the use of endotypes, phenotypes, and distinct biomarkers will replace the rough cut “one-size-fits-all” approach. From this viewpoint, we feel that the current view of the allergy/asthma link is too generalized. Asthma, as discussed above, is indeed linked to allergy from a broad perspective and especially in the pediatric age, but such oversimplifications do not appear to further serve us. Asthma has a huge heterogeneity due to individual genetic and epigenetic variability and discrete environmental exposures, which are dependent on regional characteristics, varying climatic conditions, and population distributions (7). This complexity is further increased when looking at the highly variable severity of the symptoms, which has in fact been used as a criterion for further subphenotyping of asthma (severe, mild, treatment resistant, etc). Different patients can have differing disease severities, even within the same endotype (2), wherein treatment response can also vary greatly, e.g., in allergic asthma response to medication can be influenced by both the degree of allergic reactivity and allergen exposure, and their complex interactions (2).

All this evidence suggests that sweeping generalizations in regard to the asthma/atopy link are probably inappropriate, as “In asthma we must embrace the concept of a complex endotype consisting of several sub-endotypes” (10). In conclusion, in this “basket,” that is asthma, there are several “eggs” of atopy, but a closer look will reveal several other eggs that we have yet to identify.

## Author Contributions

GG conceptualized and drafted the manuscript, conducted the literature search, and approved the final version as submitted. AM, MT, and ST assisted in drafting and literature search, critically reviewed the manuscript, and approved submission.

## Conflict of Interest Statement

The authors declare that the research was conducted in the absence of any commercial or financial relationships that could be construed as a potential conflict of interest.

## References

[1] Carr TF Bleecker E. Asthma heterogeneity and severity. World Allergy Organ J (2016) 9(1):41. 10.1186/s40413-016-0131-2 27980705 PMC5129643

[2] Lotvall J Akdis CA Bacharier LB Bjermer L Casale TB Custovic A Asthma endotypes: a new approach to classification of disease entities within the asthma syndrome. J Allergy Clin Immunol (2011) 127(2):355–60. 10.1016/j.jaci.2010.11.037 21281866

[3] Ross RN Nelson HS Finegold I. Effectiveness of specific immunotherapy in the treatment of asthma: a meta-analysis of prospective, randomized, double-blind, placebo-controlled studies. Clin Ther (2000) 22(3):329–41. 10.1016/S0149-2918(00)80037-5 10963287

[4] Gibson PG Simpson JL Saltos N. Heterogeneity of airway inflammation in persistent asthma: evidence of neutrophilic inflammation and increased sputum interleukin-8. Chest (2001) 119(5):1329–36. 10.1378/chest.119.5.1329 11348936

[5] Koczulla AR Vogelmeier CF Garn H Renz H New concepts in asthma: clinical phenotypes and pathophysiological mechanisms. Drug Discov Today (2017) 22(2):388–96. 10.1016/j.drudis.2016.11.008 27867084

[6] Agache I Akdis C Jutel M Virchow JC. Untangling asthma phenotypes and endotypes. Allergy (2012) 67(7):835–46. 10.1111/j.1398-9995.2012.02832.x 22594878

[7] Agache I Akdis CA. Endotypes of allergic diseases and asthma: an important step in building blocks for the future of precision medicine. Allergol Int (2016) 65(3):243–52. 10.1016/j.alit.2016.04.011 27282212

[8] Haldar P Pavord ID Shaw DE Berry MA Thomas M Brightling CE Cluster analysis and clinical asthma phenotypes. Am J Respir Crit Care Med (2008) 178(3):218–24. 10.1164/rccm.200711-1754OC 18480428 PMC3992366

[9] Woodruff PG Modrek B Choy DF Jia G Abbas AR Ellwanger A T-helper type 2-driven inflammation defines major subphenotypes of asthma. Am J Respir Crit Care Med (2009) 180(5):388–95. 10.1164/rccm.200903-0392OC 19483109 PMC2742757

[10] Agache I Sugita K Morita H Akdis M Akdis CA. The complex type 2 endotype in allergy and asthma: from laboratory to bedside. Curr Allergy Asthma Rep (2015) 15(6):29. 10.1007/s11882-015-0529-x 26141574

[11] Moore WC Meyers DA Wenzel SE Teague WG Li H Li X Identification of asthma phenotypes using cluster analysis in the Severe Asthma Research Program. Am J Respir Crit Care Med (2010) 181(4):315–23. 10.1164/rccm.200906-0896OC 19892860 PMC2822971

[12] Gauvreau GM Boulet LP Cockcroft DW Fitzgerald JM Carlsten C Davis BE Effects of interleukin-13 blockade on allergen-induced airway responses in mild atopic asthma. Am J Respir Crit Care Med (2011) 183(8):1007–14. 10.1164/rccm.201008-1210OC 21057005

[13] Wenzel S Ford L Pearlman D Spector S Sher L Skobieranda F Dupilumab in persistent asthma with elevated eosinophil levels. N Engl J Med (2013) 368(26):2455–66. 10.1056/NEJMoa1304048 23688323

[14] Kau AL Korenblat PE. Anti-interleukin 4 and 13 for asthma treatment in the era of endotypes. Curr Opin Allergy Clin Immunol (2014) 14(6):570–5. 10.1097/ACI.0000000000000108 25159182 PMC4374802

[15] Chung KF. Asthma phenotyping: a necessity for improved therapeutic precision and new targeted therapies. J Intern Med (2016) 279(2):192–204. 10.1111/joim.12382 26076339

[16] Castro M Mathur S Hargreave F Boulet LP Xie F Young J Reslizumab for poorly controlled, eosinophilic asthma: a randomized, placebo-controlled study. Am J Respir Crit Care Med (2011) 184(10):1125–32. 10.1164/rccm.201103-0396OC 21852542

[17] Ortega HG Liu MC Pavord ID Brusselle GG FitzGerald JM Chetta A Mepolizumab treatment in patients with severe eosinophilic asthma. N Engl J Med (2014) 371(13):1198–207. 10.1056/NEJMoa1403290 25199059

[18] Walsh GM. Mepolizumab-based therapy in asthma: an update. Curr Opin Allergy Clin Immunol (2015) 15(4):392–6. 10.1097/ACI.0000000000000183 26110690

[19] Darveaux J Busse WW Biologics in asthma – the next step toward personalized treatment. J Allergy Clin Immunol Pract (2015) 3(2):152–160; quiz 161. 10.1016/j.jaip.2014.09.014 25754716 PMC4774509

[20] Agache IO. From phenotypes to endotypes to asthma treatment. Curr Opin Allergy Clin Immunol (2013) 13(3):249–56. 10.1097/ACI.0b013e32836093dd 23587683

[21] Hanania NA Wenzel S Rosen K Hsieh HJ Mosesova S Choy DF Exploring the effects of omalizumab in allergic asthma: an analysis of biomarkers in the EXTRA study. Am J Respir Crit Care Med (2013) 187(8):804–11. 10.1164/rccm.201208-1414OC 23471469

[22] Wesolowska-Andersen A Seibold MA. Airway molecular endotypes of asthma: dissecting the heterogeneity. Curr Opin Allergy Clin Immunol (2015) 15(2):163–8. 10.1097/ACI.0000000000000148 25961390 PMC4857192

[23] Martin PE Matheson MC Gurrin L Burgess JA Osborne N Lowe AJ Childhood eczema and rhinitis predict atopic but not nonatopic adult asthma: a prospective cohort study over 4 decades. J Allergy Clin Immunol (2011) 127(6):1473–9.e1. 10.1016/j.jaci.2011.02.041 21458851

[24] Al-Ramli W Prefontaine D Chouiali F Martin JG Olivenstein R Lemiere C T(H)17-associated cytokines (IL-17A and IL-17F) in severe asthma. J Allergy Clin Immunol (2009) 123(5):1185–7. 10.1016/j.jaci.2009.02.024 19361847

[25] Chien JW Lin CY Yang KD Lin CH Kao JK Tsai YG. Increased IL-17A secreting CD4+ T cells, serum IL-17 levels and exhaled nitric oxide are correlated with childhood asthma severity. Clin Exp Allergy (2013) 43(9):1018–26. 10.1111/cea.12119 23957337

[26] Dejager L Dendoncker K Eggermont M Souffriau J Van Hauwermeiren F Willart M Neutralizing TNFalpha restores glucocorticoid sensitivity in a mouse model of neutrophilic airway inflammation. Mucosal Immunol (2015) 8(6):1212–25. 10.1038/mi.2015.12 25760421

[27] Simpson JL Grissell TV Douwes J Scott RJ Boyle MJ Gibson PG. Innate immune activation in neutrophilic asthma and bronchiectasis. Thorax (2007) 62(3):211–8. 10.1136/thx.2006.061358 16844729 PMC2117164

[28] Cosmi L Maggi L Santarlasci V Capone M Cardilicchia E Frosali F Identification of a novel subset of human circulating memory CD4(+) T cells that produce both IL-17A and IL-4. J Allergy Clin Immunol (2010) 125(1):222–30.e1–4. 10.1016/j.jaci.2009.10.012 20109749

[29] Irvin C Zafar I Good J Rollins D Christianson C Gorska MM Increased frequency of dual-positive TH2/TH17 cells in bronchoalveolar lavage fluid characterizes a population of patients with severe asthma. J Allergy Clin Immunol (2014) 134(5):1175–86.e7. 10.1016/j.jaci.2014.05.038 25042748 PMC4254017

[30] Hesselmar B Enelund AC Eriksson B Padyukov L Hanson LA Aberg N. The heterogeneity of asthma phenotypes in children and young adults. J Allergy (Cairo) (2012) 2012:163089. 10.1155/2012/163089 22577403 PMC3332210

[31] Wenzel S Severe asthma: from characteristics to phenotypes to endotypes. Clin Exp Allergy (2012) 42(5):650–8. 10.1111/j.1365-2222.2011.03929.x 22251060

[32] Wenzel SE Asthma phenotypes: the evolution from clinical to molecular approaches. Nat Med (2012) 18(5):716–25. 10.1038/nm.2678 22561835

[33] Szefler SJ Phillips BR Martinez FD Chinchilli VM Lemanske RF Strunk RC Characterization of within-subject responses to fluticasone and montelukast in childhood asthma. J Allergy Clin Immunol (2005) 115(2):233–42. 10.1016/j.jaci.2004.11.014 15696076

[34] Martinez FD Wright AL Taussig LM Holberg CJ Halonen M Morgan WJ. Asthma and wheezing in the first six years of life. The group Health Medical Associates. N Engl J Med (1995) 332(3):133–8. 10.1056/NEJM199501193320301 7800004

[35] Guibas GV Makris M Papadopoulos NG. Acute asthma exacerbations in childhood: risk factors, prevention and treatment. Expert Rev Respir Med (2012) 6(6):629–38. 10.1586/ers.12.68 23234449

[36] Krishnamoorthy N Khare A Oriss TB Raundhal M Morse C Yarlagadda M Early infection with respiratory syncytial virus impairs regulatory T cell function and increases susceptibility to allergic asthma. Nat Med (2012) 18(10):1525–30. 10.1038/nm.2896 22961107 PMC3641779

[37] Fitzpatrick AM Teague WG Meyers DA Peters SP Li X Li H Heterogeneity of severe asthma in childhood: confirmation by cluster analysis of children in the National Institutes of Health/National Heart, Lung, and Blood Institute Severe Asthma Research Program. J Allergy Clin Immunol (2011) 127(2):382–9.e1–13. 10.1016/j.jaci.2010.11.015 21195471 PMC3060668

[38] Moore WC Bleecker ER Curran-Everett D Erzurum SC Ameredes BT Bacharier L Characterization of the severe asthma phenotype by the national heart, lung, and blood institute’s severe asthma research program. J Allergy Clin Immunol (2007) 119(2):405–13. 10.1016/j.jaci.2006.11.639 17291857 PMC2837934

[39] Henderson J Granell R Heron J Sherriff A Simpson A Woodcock A Associations of wheezing phenotypes in the first 6 years of life with atopy, lung function and airway responsiveness in mid-childhood. Thorax (2008) 63(11):974–80. 10.1136/thx.2007.093187 18678704 PMC2582336

[40] Savenije OE Granell R Caudri D Koppelman GH Smit HA Wijga A Comparison of childhood wheezing phenotypes in 2 birth cohorts: ALSPAC and PIAMA. J Allergy Clin Immunol (2011) 127(6):1505–12.e14. 10.1016/j.jaci.2011.02.002 21411131

[41] Spycher BD Silverman M Brooke AM Minder CE Kuehni CE. Distinguishing phenotypes of childhood wheeze and cough using latent class analysis. Eur Respir J (2008) 31(5):974–81. 10.1183/09031936.00153507 18216047

[42] Spycher BD Silverman M Pescatore AM Beardsmore CS Kuehni CE. Comparison of phenotypes of childhood wheeze and cough in 2 independent cohorts. J Allergy Clin Immunol (2013) 132(5):1058–67. 10.1016/j.jaci.2013.08.002 24075230

[43] Sears MR Johnston NW. Understanding the September asthma epidemic. J Allergy Clin Immunol (2007) 120(3):526–9. 10.1016/j.jaci.2007.05.047 17658590 PMC7172191

[44] Shaheen SO Changing patterns of childhood infection and the rise in allergic disease. Clin Exp Allergy (1995) 25(11):1034–7. 10.1111/j.1365-2222.1995.tb03248.x 8581834

[45] Jackson DJ Gangnon RE Evans MD Roberg KA Anderson EL Pappas TE Wheezing rhinovirus illnesses in early life predict asthma development in high-risk children. Am J Respir Crit Care Med (2008) 178(7):667–72. 10.1164/rccm.200802-309OC 18565953 PMC2556448

[46] Skloot GS. Asthma phenotypes and endotypes: a personalized approach to treatment. Curr Opin Pulm Med (2016) 22(1):3–9. 10.1097/MCP.0000000000000225 26574717

[47] Ray A Oriss TB Wenzel SE. Emerging molecular phenotypes of asthma. Am J Physiol Lung Cell Mol Physiol (2015) 308(2):L130–40. 10.1152/ajplung.00070.2014 25326577 PMC4338947

[48] Coleman JM Naik C Holguin F Ray A Ray P Trudeau JB Epithelial eotaxin-2 and eotaxin-3 expression: relation to asthma severity, luminal eosinophilia and age at onset. Thorax (2012) 67(12):1061–6. 10.1136/thoraxjnl-2012-201634 23015684 PMC3652589

[49] Boulet LP. Asthma in the elderly patient. Asthma Res Pract (2016) 2:3. 10.1186/s40733-015-0015-1 27965771 PMC5142394

[50] Moorman JE Mannino DM. Increasing U.S. asthma mortality rates: who is really dying? J Asthma (2001) 38(1):65–71. 10.1081/JAS-100000023 11256556

[51] Kim TB Jang AS Kwon HS Park JS Chang YS Cho SH Identification of asthma clusters in two independent Korean adult asthma cohorts. Eur Respir J (2013) 41(6):1308–14. 10.1183/09031936.00100811 23060627

[52] Dixon AE Pratley RE Forgione PM Kaminsky DA Whittaker-Leclair LA Griffes LA Effects of obesity and bariatric surgery on airway hyperresponsiveness, asthma control, and inflammation. J Allergy Clin Immunol (2011) 128(3):508–15.e1–2. 10.1016/j.jaci.2011.06.009 21782230 PMC3164923

[53] Farzan S. The asthma phenotype in the obese: distinct or otherwise? J Allergy (Cairo) (2013) 2013:602908. 10.1155/2013/602908 23878548 PMC3708411

[54] Colak Y Afzal S Lange P Nordestgaard BG. Obese individuals experience wheezing without asthma but not asthma without wheezing: a Mendelian randomisation study of 85,437 adults from the Copenhagen General Population Study. Thorax (2016) 71(3):247–54. 10.1136/thoraxjnl-2015-207379 26504195

[55] McGrath KW Icitovic N Boushey HA Lazarus SC Sutherland ER Chinchilli VM A large subgroup of mild-to-moderate asthma is persistently noneosinophilic. Am J Respir Crit Care Med (2012) 185(6):612–9. 10.1164/rccm.201109-1640OC 22268133 PMC3326288

[56] Hodge S Hodge G Simpson JL Yang IA Upham J James A Blood cytotoxic/inflammatory mediators in non-eosinophilic asthma. Clin Exp Allergy (2016) 46(1):60–70. 10.1111/cea.12634 26767492

[57] Baines KJ Simpson JL Wood LG Scott RJ Gibson PG. Transcriptional phenotypes of asthma defined by gene expression profiling of induced sputum samples. J Allergy Clin Immunol (2011) 127(1):153–60, 160.e1–9. 10.1016/j.jaci.2010.10.024 21211650

[58] Greenberger PA. Allergic bronchopulmonary aspergillosis. J Allergy Clin Immunol (2002) 110(5):685–92. 10.1067/mai.2002.130179 12417875

